# Synovial Tissue Infection with *Burkholderia fungorum*

**DOI:** 10.3201/eid2210.151114

**Published:** 2016-10

**Authors:** Shih Keng Loong, Yih Harng Soh, Nur Hidayana Mahfodz, Jefree Johari, Sazaly AbuBakar

**Affiliations:** University of Malaya, Kuala Lumpur, Malaysia (S.K. Loong, N.H. Mahfodz, J. Johari, S. AbuBakar);; University Malaya Medical Centre, Kuala Lumpur (Y.H. Soh)

**Keywords:** Burkholderia, Malaysia, infectious disease, synovial tissue, multilocus sequence typing, bacteria, Burkholderia fungorum

**To the Editor:** The genus *Burkholderia*, first proposed in 1992, has now expanded to consist of many species (*1*). Bacteria of this genus exhibit extensive ubiquity; they have been isolated from human, animal, and environmental sources (*1*,*2*). Although rare, *Burkholderia fungorum* has been implicated in several human infections (*2*–*4*). We isolated *B. fungorum* from the synovial tissue of a patient’s knee.

In February 2011, a 26-year-old man had a meniscal tear on the left knee but deferred surgery. Two years later, the same knee was injured after a fall. On November 1, 2013, the patient underwent anterior cruciate ligament reconstruction. On November 28, 2013, he underwent arthroscopy because of decreased range of motion in the knee. The patient did not have a fever, and leukocyte count and erythrocyte sedimentation rate were within reference range. The patient was obese but did not have diabetes, hypertension, or any autoimmune disease.

Arthroscopy indicated that the anterior cruciate ligament graft was intact. However, the synovial fluid was turbid, and synovial tissue infection was suspected. A synovial tissue biopsy was sent to the microbiology laboratory for culture. Test results for HIV, hepatitis B, and hepatitis C infection were negative. Fungal culture was also negative. After the tissue biopsy sample was incubated on blood agar for 48 h at an ambient temperature of 37°C, 2 different colonies grew; both showed big, yellow colonies of gram-negative rods. Vitek 2 (bioMérieux, Marcy l’Etoile, France) identified the isolates as *Pseudomonas fluorescens* (91% probability) and *Brucella melitensis* (94% probability). Because of the possible *B. melitensis* infection, all excess specimens and agar plates were sealed and discarded after being autoclaved. Later DNA amplification and sequencing of the bacterial 16S rDNA identified the isolates previously identified as *P. fluorescens* and *B. melitensis* as *Mesorhizobium amorphae* and *Burkholderia fungorum* (isolate BF370), respectively. The 16S rDNA sequences of BF370 (GenBank accession no. LN868266) displayed 99% similarity to *B. fungorum* strain DBT1 (GenBank accession no. HM113360).

Disk-diffusion tests performed according to the Clinical and Laboratory Standards Institute interpretive standards for *Burkholderia cepacia* (*5*) revealed that BF370 was sensitive to ceftazidime, meropenem, and trimethoprim/sulfamethoxazole. Additional characterization of BF370 with multilocus sequence typing (*6*) revealed a novel sequence type, 868. Novel alleles were found for 7 loci: *atpD* (allele 347), *gltB* (allele 409), *gyrB* (allele 609), *recA* (allele 367), *lepA* (allele 418), *phaC* (allele 319), and *trpB* (allele 406). Intravenous ampicillin/sulbactam (1.5 g, 3×/d) was administered to the patient from November 29, 2013, through December 13, 2013. No pus was observed at the wound, and the patient was afebrile. He did not report discomfort, and physiotherapy commenced on day 8 after surgery.

Initial identification of the isolate as *B. melitensis* raised concern for possible laboratory transmission of this pathogen (*7*). The possibility of finding brucellae in the synovial tissue was not unexpected because *Brucella* spp. have been reported to cause joint infections (*8*). However, 16S rDNA sequencing confirmed the identity of the isolate as *B. fungorum*. This different finding suggests that commercial phenotypic identification system could sometimes be unreliable. The isolation of *B. fungorum* from the synovial tissue of this patient, however, is similar to an earlier case of leg tissue infection in a girl (*3*). A striking similarity shared between the isolates was their antimicrobial drug susceptibility profiles; each isolate was susceptible to ceftazidime, meropenem, and trimethoprim/sulfamethoxazole. A literature search revealed that *B. fungorum* had first been recovered from vaginal secretions and cerebrospinal spinal fluid of 2 women (*2*). However, no clinical information was presented, leading to the absence of clinical descriptions of the infections.

Recently, *B. fungorum* was detected in a granuloma specimen from a 26-year-old patient (*4*). The bacteria were suspected to have been transmitted to the patient through a rose thorn puncture. Swelling did not appear until 3 years after the initial puncture, and the patient did not report pain or fever and was otherwise healthy (*4*). Although the clinical implication of *B. fungorum* infection remained unclear, recent cases (*3**,**4*) seem to suggest that the bacteria can induce fever in young children and has a lengthy incubation period before erupting. 

We suspect that the patient reported here acquired *B. fungorum* during his initial injury, perhaps from the environment. The concurrent isolation of *M. amorphae*, a plant bacterium (*9*), is consistent with possible environmental origin of the infection. Our observations and those reported earlier (*4*) suggest the ability of *B. fungorum* to act as a slowly replicating opportunistic pathogen associated with tissue injuries. The slow replication rate of *B. fungorum* presumably mimics the growth characteristic of *Burkholderia mallei*, essential for the bacterium’s survival, proliferation, and evasion of host adaptive immune responses (*10*). Our findings suggest that an approach combining culture, 16S rDNA sequencing, and multilocus sequence typing be considered for the accurate identification of uncommon bacterial infection.
